# Historical development of the htasialink network and its key determinants of success

**DOI:** 10.1017/S0266462318000223

**Published:** 2018-06-18

**Authors:** Yot Teerawattananon, Karlena Luz, Chalarntorn Yothasmutra, Raoh-Fang Pwu, Jeonghoon Ahn, Asrul Akmal Shafie, Kalipso Chalkidou, Sripen Tantivess, Benjarin Santatiwongchai, Waranya Rattanavipapong, Saudamini Dabak

**Affiliations:** 1 Health Intervention and Technology Assessment Program (HITAP), Thailand Saw Swee Hock School of Public Health, National University of Singapore; 2 Health Intervention and Technology Assessment Program (HITAP); 3 National Hepatitis C Program Office, Ministry of Health and Welfare, Taiwan; 4 Ewha Womans University, South Korea; 5 Discipline of Social & Administrative Pharmacy, Universiti Sains Malaysia; 6 Department of Epidemiology and Infectious Diseases, School of Public Health, Imperial College London & Center for Global Development, USA; 7 Health Intervention and Technology Assessment Program (HITAP)

**Keywords:** Health technology assessment, Asia, Organization and administration, Inter-sectoral collaboration, Achievement

## Abstract

**Objectives:**

The aim of this study was to describe the historical development of the HTAsiaLink network, draw lessons for other similar initiatives globally, and to analyze key determinants of its success and challenges for its future development.

**Methods:**

This study is based on the collective and direct experiences of the founding members of the HTAsiaLink Network. Data were collected from presentations they made at various international forums and additional information was reviewed. Data analysis was done using the framework developed by San Martin-Rodriguez et al.

**Results and Conclusions:**

HTAsiaLink is a network of health technology assessment (HTA) agencies in Asia established in 2011 with the aim of strengthening individual and institutional HTA capacity, reducing duplication and optimizing resources, transfer and sharing of HTA-related lessons among members, and beyond. During its 6 years, the network has expanded, initiating several capacity building activities and joint-research projects, raising awareness of the importance of HTA within the region and beyond, and gaining global recognition while establishing relationships with other global networks. The study identifies the determinants of success of the collaboration. The systemic factors include the favorable outlook toward HTA as an approach for healthcare priority setting in countries with UHC mandates. On organizational factors, the number of newly established HTA agencies in the region with similar needs for capacity building and peer-to-peer support was catalytic for the network development. The interactional aspects include ownership, trust, and team spirit among network members. The network, however, faces challenges notably, financial sustainability and management of the expanded network.

Health technology assessment (HTA) can help inform resource allocation, including the selection of healthcare benefit packages and essential medicines lists (1–6). Unfortunately, Asia and other regions of the Global South lack the capacity for conducting **HTA** due to several factors, including, but not limited to, lack of awareness, lack of local epidemiological data, disjointed efforts in research, and the late introduction of the field of pharmacoeconomics in the 1990s in Asia (7;8). Collaboration can offer several benefits to overcome these obstacles such as information and knowledge sharing, increase of social capital, and innovation to advance the field of **HTA** (5;9–12).

In 2011, the HTAsiaLink Network was officially established as a collaborative network for HTA agencies in Asia. Its establishment marked a major step in the diffusion of HTA in the region. The network began with three agencies who were interested in setting up a collaborative platform for mutual benefit. Over the years, the network grew in membership as well as in terms of the depth and breadth of economic and health systems research conducted by network members within the region. The network’s functions have evolved from being a platform for sharing research findings to becoming a vehicle for sharing awareness about the usefulness of HTA evidence in priority setting. The network has not only been involved in strengthening the capacities of countries that currently have expertise in HTA but also introducing HTA to countries where it is a nascent field and has not yet been recognized as a tool for policy making.

HTAsiaLink’s historical development offers insights on how collaborations can nurture new initiatives in different countries, how they can benefit HTA development in countries and lessons on developing a regional HTA network where most countries are low- and middle-income countries (LMICs). In this article, we aim to examine the key determinants that allow and encourage regional collaboration among HTA agencies in the HTAsiaLink network. This study is based on the collective and direct experiences of the founding members of the HTAsiaLink network.

Data were collected from presentations made by founding members at various international forums hosted by Health Technology Assessment International (HTAi), International Society for Pharmacoeconomics and Outcome Research (ISPOR), Asia Pacific Economic Cooperation (APEC), World Health Organization (WHO), and Prince Mahidol Award Foundation. Additional information available on the HTAsiaLink Web site was reviewed. The data were then analyzed by the authors from HITAP using the framework developed by San Martin-Rodriguez et al. (13), and the draft was shared with other authors for multiple rounds of review until all authors were satisfied with the study.

The framework proposed by San Martin-Rodriguezestablished in 1985 as et al. is one of the few that offers a simple and powerful tool to understand the determinants of a successful collaboration. The framework describes the three main determinants that contribute to the success of a collaboration, namely systemic (elements outside the organization which are components of social, cultural, educational, and professional systems), organizational (attributes of organizations that define the work environment of the network, such as its structure, philosophy, team resources, administrative support, as well as communication and coordination mechanisms), and interactional (components of interpersonal relationships among the collaborators such as willingness to collaborate, mutual trust, respect, and communication).

## FINDINGS

### Systemic Factors

Although several HTA collaborations at the global and regional levels existed before its formation, HTAsiaLink is the only HTA network initiated by actors within the region. Collaborations that function at the global level include, for example, the Society for Medical Decision Making (SMDM) established in 1979, the International Network of Agencies for Health Technology Assessment (INAHTA) established in 1993, the ISPOR established in 1995, and the HTAi established in 1985 as ISTAHC (12). Regionally, collaborations such as the HTAnetAsia of ISPOR, were attended by several individuals in the region. These existing collaborations were managed by organizations outside the region, either from Europe or North America and were not necessarily responsive to the unique demands of newly established HTA agencies in Asia which are described hereafter.

Despite the existing collaborations being informative and useful for the development of HTA in Asia, some barriers inhibited collaboration. First, global HTA networks request significant membership fees and registration fees to attend their conferences or meetings. These conferences are typically held in different cities, in some cases, different continents, which can incur substantial travelling costs to participants from LMICs. That said, many networks including HTAi and ISPOR regularly organize regional and country meetings. While key staff from organizations in LMICs can participate, the fees, coupled with the cost of traveling for new and young staff, who are most in need of the information shared and the training, can be prohibitive. Second, the activities of these networks focus on conducting annual or biannual conferences and sharing information rather than supporting technical activities. Although technical training is offered, these usually take place alongside the conferences. Rarely are they able to facilitate collaboration on primary work, apart from questionnaire surveys of members. Third, the existing networks, with the exception of INAHTA, consist of individual and organizational members from both public and private sectors, resulting in loosely formed networks with divergent interests. This may hinder discussion and support on sensitive policy issues that is needed for HTA agencies.

One of the systemic factors that strongly contributed to the establishment of HTAsiaLink was the demand for HTA development in Asian countries committed to Universal Healthcare Coverage (UHC). Half of the fourteen settings in the region namely, Bhutan, Japan, Korea, Malaysia, Singapore, Taiwan, and Thailand have already achieved UHC. Although other settings such as Indonesia, the Philippines, and Vietnam are yet to achieve UHC, they have shown strong commitment to and recognize the need for evidence-informed priority setting as demonstrated by government legislations (14–17). Table 1 shows that all fourteen settings commit significant resources to healthcare, especially public resources in terms of percentage of government expenditure. Moreover, these countries also face healthcare challenges due to an increasingly aging population resulting in a higher demand for healthcare.

**Table 1:** HTAsiaLink Members by Setting

Sites	Year of achieving UHC	THE as % of GDP	GHB as % of Government budget(8)	HTAsiaLink Members
Australia	N/A	9.4	18.7	• HealthPACT (2013) • ASERNIP-S (2014) • U of Sydney (2014)
Bhutan	N/A	3.6	6.6	• EMTD (2013)
China	2020	5.4	12.5	• CNHDRC (2012) • TJAB (2012)
Taiwan	1995	6.9	19.8	• CDE (2010) • NTU (2015) • Taipei Medical U (2015)
Indonesia	2019	3.44	6.9	• MoH (2014)
Japan	N/A	10.2	20	• NIPH (2012)
Kazakhstan	N/A	4.4	10.9	• MoH (2015)
New Zealand	N/A	11	20.5	• NHC (2015)
Malaysia	1980s^(9)^	4.75	5.8	• MaHTAS (2013) • USM (2010) • PSD (2012)
Mongolia	N/A	4.7	10.3	• Leading Researchers (2015)
Philippines	N/A	4.7	8.5	• NCPAM (2013)
Republic of Korea	1988	6.8	13.6	• NECA (2010)
Singapore	N/A	4.9	12.5	• MoH (2010) • NUS (2013) • EHA (2014) • ACE (2015) • HSRI (2015) • AMRI (2015) • Saw Swee Hock (2016)
Sri Lanka	N/A	3.5	5.3	• U-Colombo (2016)
Thailand	2002	4.5	14.2	• HITAP (2010) • IHPP (2014)
Viet Nam	2020	6.0	9.5	• HSPI (2014)

*Note*. Unreferenced statistics were based on information given by the area authors. For sites that have not yet achieved UHC, the year presented is set by the government. Sources: World Bank (2015); WHO (2015); Savedoff WD, Smith AL (2011); National Statistics Republic of China (2014).

GDP, gross domestic product; GHB, government health budget; THE, total health expenditure; UHC, universal health coverage.

Given that HTA is regarded as a policy and technical tool to support governments in setting health priorities under limited resources, HTAsiaLink can be seen as a platform for learning that contributes to awareness, acceptance and adoption of HTA in settings that need it most. The structure of the network (to be described below) also contributes to creating a social system where the network values and respects equality among members for decision making. This gives all members the power to steer the management of the network, thereby encouraging their involvement as compared with other networks.

### Organizational Factors

The mission of the network is to address the following issues: (i) strengthening individual and institutional capacity in HTA research and integration of HTA evidence into policy decisions for the public good; (ii) avoiding duplication especially in reviewing safety and clinical efficacy of vaccines and medicines for HTA, facilitating learning, reducing wasteful resource use, and enhancing efficiency at organizational level through collaborative activities among the network; and (iii) fulfilling the need for transferring and sharing HTA-related lessons across countries and organizations in Asia and beyond (18).

The overarching philosophy behind the network, as outlined by its mission, is to focus on capacity development and indepth information sharing and is one of the key organizational factors that has facilitated its growth. This philosophy addresses the fundamental need of the organizations in the region and as such affects their willingness to actively contribute to the collaboration. All members of HTAsiaLink voluntarily contribute to the network activities; for example, NECA and HITAP commit their own resources to serve as the network’s secretariat and the newsletter’s editorial team, respectively. Once a network member needs policy relevant information from other countries, they can use the channels opened by this network to request information informally and other network members can voluntarily provide this information without any obligation. This is a time and cost-efficient method of acquiring information and enhances the team spirit among network members.

Over the past 6 years, there has been an increased interest in becoming a member of the HTAsiaLink network, including by HTA agencies and research units in Australia and New Zealand. In 2015, the by-laws of the network were amended to accept organizations from the Oceania region with full membership as shown in Table 1. The network’s membership has gradually grown due to continuous engagement and activities as well as the low barriers to entry. Currently, there are thirty members from sixteen settings. At the global level, several academic publications have recognized HTAsiaLink (2;19;20). Additionally, associated organizational members were accepted by the network to allow for the participation of actors outside Asia due to their interest or active involvement in the region. These organizations include NICE International (2011), PRICELESS, University of Witwatersrand (2017), Global Health and Development Group, and Imperial Collage (2017). Furthermore, partnerships with other HTA agencies have been forged by signing a memorandum of understanding (MOU) with INAHTA in 2016 to mutually support each other’s activities. MOUs with other networks, namely, EuroScan and HTAi, are under discussion.

The management of the network, including its organizational structure, governance and activities is not only aligned with members’ values but also enhances trust among them. The flexibility in terms of management, reliance on voluntary contributions, and the disallowance of interference by commercial interests are among the organizational factors that have underpinned its value for the network members. The noninvolvement of commercial interests is reflected in the increasing number of sensitive policy issues being consulted among members as well as the willingness to initiate and participate in joint projects.

One of the network’s main activities is an Annual HTAsialink Conference that started in 2012 in Thailand. In the following years, the annual conferences continued and were hosted by different volunteer local organizers in Malaysia, China, Taiwan, Singapore, and Vietnam. The conference focuses on the development of HTA capacity and networking among junior staff of HTAsiaLink organizational members and does not charge any membership or conference fee for participants. To allow for open discussion of issues regarded as sensitive to public HTA agencies, the conference does not allow participants from the healthcare products industry to join.

Local organizers of conferences also commit their own resources or mobilize additional funding support to organize the conference. Members pay for their own travel and accommodation. Organizations such as HTAi, International Decision Support Initiative (iDSI), INAHTA, Rockefeller Foundation, and WHO also provide funding support. Other activities are either funded by the organization that initiated the collaboration or by agencies that agreed to participate in the collaborative activity.

**Figure 1:**
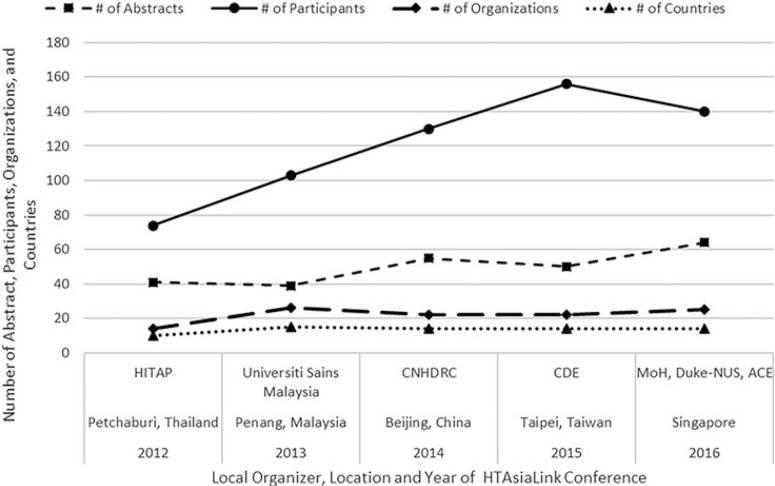
Conference progression.

As illustrated in Figure 1, each year the size of the conference has increased in terms of the number of included abstracts, participants, involvement of organizations, and represented countries. The conferences generally have three main components: preconference training workshops, plenaries delivered by policy makers and experts, and oral presentations by junior researchers. Preconference workshops on topics such as Critiquing Manufacturers’ Economic Models (21), allow global experts to share experience about technical or policy issues related to the use of HTA by public authorities. Plenaries, for example one titled, Leaders Forum-Highlights from High-Level Decision Makers on HTA for UHC (22), are organized for regional and global experts to share issues of interest related to the theme of the conference. The conference is primarily dedicated to research presentations made by junior researchers. The scope of the presentations is divided into two parts, health systems, policy research and economic evaluations. Unlike other HTA conferences where senior experts monopolize the key sessions, these experts are instead assigned to provide constructive comments on research presentations by junior staff from HTAsiaLink’s member organizations to nurture HTA capacity in the region.

**Figure 2:**
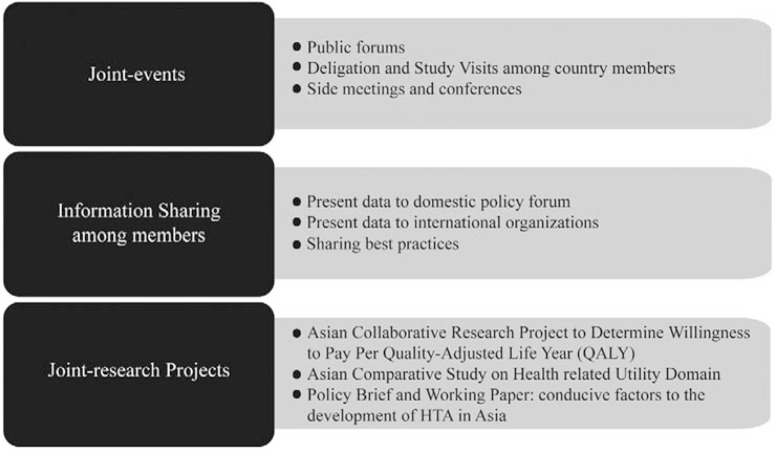
Joint collaborations.

Organizational members are also encouraged to initiate joint activities, including research projects as a way of building-up networking capacity in conducting policy-relevant primary research across different settings as illustrated in Figure 2. For example, the first joint research was conducted on the Asian Collaborative Research Project to Determine Willingness to Pay Per Quality-Adjusted Life Year (QALY), which included primary data collection in four settings using a standard tool jointly developed by the group. Additional work included a study to determine factors influencing health-related utility among populations in four Asian settings leading to the development of a working paper on conducive factors to the development of HTA in Asia (23–25). Ongoing projects include the Guide for Economic Analysis and Research (GEAR) database which is co-founded by HTAsiaLink alongside other partners. It is a Web-based resource designed to aid in research and analysis of economic evaluations in the context of LMICs (26).

Another unique activity of the network is informal consultation on policy sensitive issues, facilitated by its secretariat. These activities help boost HTA in member countries struggling to foster HTA. Most topics consulted on relate to urgent policy issues faced by organizational members who want to get information from other HTA agencies in the region. This has been perceived as a successful mechanism that members appreciate. Examples of the topics consulted include availability of evaluations on the robotic-assisted surgery or da Vinci surgery requested by Chinese authorities and the situation on coverage decisions on glucosamine sulphate for osteoarthritis requested by Thai authorities. These exchanges have helped enhance communication and build trust among the members.

### Interactional Factors

Interactional factors primarily relate to the composition of the membership. Because most of the network’s organizational members are newly established HTA agencies with relatively few experienced staff, mutual respect is acknowledged by the experienced agencies that impart knowledge and guidance and the agencies that make an effort to learn and establish HTA mechanisms in their own settings. Given that the members of the network are from the same region, share cultural attributes and common policy challenges, this encourages collaborative behavior within the network. Accordingly, leaders of founding organizational members have developed good personal relationships through close interaction in other conferences, meetings, and policy forums (27).

The creation of the network set up by key actors within the region, has contributed to overcoming the barriers for collaboration perceived by the founding members as described above.

Between HTAi Annual Conference in Dublin in June 2010 and ISPOR 4th Asia-Pacific Conference in Phuket in September 2010, discussions were on-going regarding the formation of a regional HTA collaborative. In 2011, the Center for Drug Evaluation (CDE), Taiwan, the Health Intervention and Technology Assessment Program (HITAP), Thailand, the National Evidence-based Healthcare Collaborating Agency (NECA), Korea, Universiti Sains Malaysia (USM), as well as individuals from the University of Tokyo and the Singapore Ministry of Health formally formed the HTAsiaLink Network.

Several formal and informal communication channels, such as direct email exchanges, tele-conferences, face-to-face meetings among HTAsiaLink members, board meetings, the news-letters and Web site (28), among others have allowed for frequent interaction. Currently the HTAsiaLink newsletter, produced biannually and edited by the HITAP communication team with contributions from all members, is circulated by means of traditional means during conferences and by means of electronic means among 500 subscribers in different regions of the globe. Sharing experience of each HTA agency through presentations and features on their achievements in the network newsletter can be used to build confidence of members. Interaction among junior staff during the annual conferences and through joint activities enable formation of stronger bonds. In most cases, junior staff are neophytes with regards to participating and presenting work at an international forum and sometimes, the research topics presented are similar but have been conducted in different settings. As a result, this encourages the presenters and participants to work with each other through peer learning and the sharing of best practices which in turn triggers more collaborative projects after the conference.

## IMPLICATIONS AND LESSONS LEARNED

HTAsiaLink is not only a regional collaboration in HTA or in health systems and policy research, its unique history and character also makes it a story worth sharing. Other regional HTA collaborations also exist for example, in Europe, EUnetHTA was established in 2005, and in the Americas, RedETSA was established in 2011. HTAsiaLink differs from these two networks in terms of context, organizational features, financial sources, network structure, operations, and scope of activities. And unlike these networks that have significant funding sources, such as the European Commission for EUnetHTA and the Pan American Health Organization (PAHO/WHO) and the United States Agency for International Development (USAID) for RedETSA, HTAsiaLink does not have a dedicated stream of financial support.

The identification of the requisite resources has been a challenge for scaling up and sustaining the network’s activities in the long term, however, HTAsiaLink has been able to draw on its existing resources to meet the needs of its members. HTAsiaLink’s reliance on the leadership of established agencies in the region, particularly in terms of in-kind and direct contributions of human and financial resources is a big factor for starting the network and being able to continue for this long without reliance on external funding sources or commercial sponsorship, maintaining an independent outlook based on the needs of its members. The interactional factors such as having close relationships among key members of the network, trust, willingness to collaborate, and mutual respect encourage members to contribute to the network in kind and financially given that the network does not have a set financial source, such as a grant or commercial sponsorship.

Scaling up of the network can also take the form of expanding its membership base. The inclusion of new members may, however, have an impact on the interactional factors that have been at the core of HTAsiaLink’s success. Although organizational factors such as structures, management, and certain decision-making rules are stated in the by-laws, its scope may be insufficient to handle a growing body. In practice, not all members are aware of these organizational factors, essentially because a majority of them are new members. The network also lacks formal documentation regarding certain organizational elements such as strategy and planning model, protocol on management, coordination, and communication, information management system, evaluation process based on the needs of the network. One solution may be to establish a working group on involving all members with the task of drafting a concept paper tackling these issues. Alternatively, the network may opt for a restricted membership base that can be easily managed.

With an increasing interest in HTA development in other regions of the world, newly established regional networks such as the African Health Economics and Policy Association (AfHEA) and the Regional Network of HTA in the Middle East and Mediterranean (EZcollab) have come to the fore. This study offers lessons learned for similar organizations as well as overseas development agencies, who increasingly play an active role in initiating regional and global collaboration to ensure effective partnerships.

In conclusion, as HTAsiaLink enters its 8th year, the upcoming conference to be held in May 2018 in Chiang Mai, Thailand, is expected to be the largest in terms of participants and sessions. This study analyses the determinants of success of the collaboration. One of the main systemic factors that enabled the formation of HTAsiaLink is that the countries involved had UHC mandates that necessitated explicit priority setting for developing the UHC benefits package. This facilitated the political and technical acceptance of HTA as an approach for healthcare priority setting. On organizational factors, the presence of several newly established HTA agencies in the region with similar needs for capacity building and peer-to-peer support was important. The network has been designed in response to this unique demand and to fill the gaps of existing networks emphasizing trust-based collaboration at minimum cost.

The interactional aspects of HTAsiaLink include giving power to members to initiate and respond to requests depending on their ability and willingness to do so which enhances team spirit. The exclusion of commercial interests in the network allows members to interact on the basis of trust and a common objective. There are several communication channels that allow for diffusion of knowledge, networking of staff and initiation of joint activities among members. That said, the network faces challenges including financial sustainability and management of the expanded network.
